# Risk factors for age-related macular degeneration: an umbrella analysis of systematic review and meta-analysis

**DOI:** 10.3389/fmed.2026.1878292

**Published:** 2026-06-17

**Authors:** Huaixiao Zang, Lin Han, Dong Shi

**Affiliations:** Department of Ophthalmology, The Fourth Affiliated Hospital of China Medical University, Shenyang, Liaoning, China

**Keywords:** age-related macular degeneration, eye disease, meta-analysis, risk factor, umbrella review

## Abstract

**Background:**

The objective of the current study was to conduct an umbrella review of meta-analyses to systematically assess the methodological quality, potential biases, and validity of all epidemiological evidence addressing risk factors associated with age-related macular degeneration (AMD), and to synthesize the available evidence regarding AMD risk factors.

**Methods:**

We searched PubMed, Web of Science, Embase, and the Cochrane Database of Systematic Reviews to April 2025 (last update) for systematic reviews and meta-analyses that focused on the risk factors for AMD. The methodological quality of each study was assessed independently by two reviewers using AMSTAR and the GRADE framework.

**Results:**

Following a comprehensive systematic search, a total of 53 distinct risk factors were identified, comprising 30 factors with statistically significant associations and 23 without significant associations. Based on the GRADE framework, most of the evidence was classified as low or very low quality, with three factors achieving a moderate level of evidence. Consistent associations were observed for age and smoking, as well as for certain lifestyle and systemic factors, including diet, diabetes, and hypertension. Circulating biomarkers, including carotenoids, C-reactive protein (CRP), and high-density lipoprotein cholesterol (HDL-C), were also found to be associated with AMD, although the strength of evidence varied across studies.

**Conclusion:**

This umbrella review indicates that AMD risk is associated with multiple modifiable lifestyle and systemic factors. Healthy diet and physical activity may have protective roles, while several circulating biomarkers, including carotenoids and C-reactive protein, have been identified as related to AMD risk. However, given that most of the available evidence is of low or very low certainty, these findings should be considered with caution.

**Systematic Review Registration:**

PROSPERO, CRD420251045558, URL: https://www.crd.york.ac.uk/PROSPERO/view/CRD420251045558.

## 1 Introduction

Age-related macular degeneration (AMD) is a progressive retinal disorder that affects the macula, which is responsible for central high-acuity vision (1). It is a common cause of visual impairment in older adults (1). Early and intermediate AMD may remain asymptomatic (2). In contrast, late-stage disease can lead to marked visual impairment, including central vision loss, distortion of straight lines, and reduced visual acuity (2). Early AMD is usually defined by the presence of medium-sized drusen measuring 63–125 μm, without AMD-related pigmentary abnormalities (3). Advanced AMD is identified by the presence of neovascular AMD or geographic atrophy (3). AMD mainly affects older adults, especially those aged 55 years or above (1). The global pooled prevalence of AMD in the 45–85-year age group has been estimated at 8.69% (4). With continued population aging worldwide, incident cases are expected to rise to 39.05 million for early AMD and 6.41 million for late-stage AMD by 2050 (5). This increase highlights the importance of effective prevention.

The pathogenesis of AMD involves several interacting processes, including aging, oxidative stress, complement activation, chronic inflammation, and genetic susceptibility (6–8). These processes may promote drusen formation, retinal pigment epithelium (RPE) loss, and photoreceptor degeneration in the macula (8). Environmental and systemic factors contribute to the development of AMD (2). Previous systematic reviews and meta-analyses have identified several common risk factors for AMD, including age, smoking, alcohol consumption, hypertension, and diabetes (9–12). More recent studies have suggested that kidney disease, thyroid disease, mental disorders, and air pollution may also be associated with AMD (13–17). In addition, some nutritional supplements, particularly carotenoids and omega-3 polyunsaturated fatty acids, have been linked to delayed AMD progression (18, 19).

Although AMD risk factors have been widely studied, variation in study design, exposure assessment, and findings makes it challenging to reach definitive conclusions. No comprehensive review has systematically evaluated the overall evidence for AMD-associated risk factors. Prior to developing effective prevention strategies, it is essential to rigorously examine the methodological quality, potential sources of bias, and overall validity of the existing evidence. To fill this gap, we conducted an umbrella review of published meta-analyses to integrate the current evidence on risk factors for AMD.

## 2 Methods and analysis

### 2.1 Design and registration

This systematic review rigorously identified, extracted, and analyzed data from published systematic reviews and meta-analyses investigating risk factors for AMD, with methodological processes strictly adhering to PRISMA guidelines (20). This study was conducted in adherence to established methodological frameworks, including the JBI Manual for Evidence Synthesis of Umbrella Reviews and the Cochrane Handbook for Systematic Reviews (21, 22). To ensure methodological transparency and protocol fidelity, the study underwent prospective registration with PROSPERO (registration ID: CRD420251045558).

### 2.2 Eligibility criteria

Eligible studies included systematic reviews and meta-analyses assessing risk factors for AMD in all demographic groups (including any ethnicity or sex) and geographical settings. When a meta-analysis evaluated two or more risk factors, data for each risk factor were extracted separately. When multiple meta-analyses investigating identical risk factors appeared with a gap of over 24 months, priority was given to the latest study for data extraction and analysis. When multiple meta-analyses addressing identical risk factors were conducted within a 24-month timeframe, selection criteria prioritized those incorporating the largest number of prospective cohort studies. When equivalent numbers of prospective cohort studies were present across eligible meta-analyses, methodological quality assessment using AMSTAR scoring determined final inclusion. Additionally, in cases where the most recent meta-analysis lacked a dose–response analysis, while other eligible analyses provided such an assessment, both types of studies were included in the data extraction process. Studies not published in English and preclinical investigations involving animal models or cell culture systems were excluded.

### 2.3 Population

In this analysis, we evaluated systematic reviews and meta-analyses assessing factors associated with AMD risk. Primary studies analyzed within these studies were required to address risk factors associated with elevated or attenuated AMD risk.

### 2.4 Exposure

Systematic reviews and meta-analyses investigating at least one AMD-associated risk factor (including personal characteristics and circulating biomarkers, environmental factors, lifestyle, underlying diseases, and pharmacological exposures) were included. The magnitude of association was quantified using odds ratios (ORs), relative risks (RRs), or hazard ratios (HRs) with corresponding 95% confidence intervals (CIs).

### 2.5 Study designs

We included only studies analyzing risk factors for AMD across all ethnicities, sexes, demographic groups, and geographic regions. Eligible reviews were required to provide a detailed description of their methodology, including a clearly reported literature search process together with predefined study selection standards, evaluation of study quality, assessment of results, analytic approaches, and interpretation criteria. The primary studies incorporated into these reviews comprised prospective and retrospective cohort studies, case–control studies, and cross-sectional investigations.

### 2.6 Information sources

We conducted a comprehensive search of PubMed, Web of Science, Embase, and the Cochrane Database of Systematic Reviews, covering all records from database inception to April 2025 (latest update), to identify systematic reviews and meta-analyses of interventional and observational studies. Furthermore, a manual screening of the bibliographies of all eligible studies was performed to identify potentially relevant articles fulfilling the predefined selection criteria.

### 2.7 Search strategy

A systematic database search was performed using MeSH terms together with relevant keywords and free-text expressions associated with AMD risk factors, in accordance with SIGN recommendations. The PubMed search strategy is described below, and the complete strategies for all databases are available in Supplementary Table S1.

The search incorporated the following terms: Macular Degeneration, Age-Related Macular Degeneration, Age Related Macular Degeneration, Age-Related Macular Degenerations, Age-Related Maculopathies, Age Related Maculopathies, Age-Related Maculopathy, Age Related Maculopathy, AMD, Risk Factors, Risk Factor, Population at Risk, Populations at Risk, Risk Scores, Risk Score, Risk Factor Scores, Risk Factor Score, Health Correlates, Social Risk Factors, Social Risk Factor, systematic review, meta-analysis.

### 2.8 Study selection

**Figure 1:**
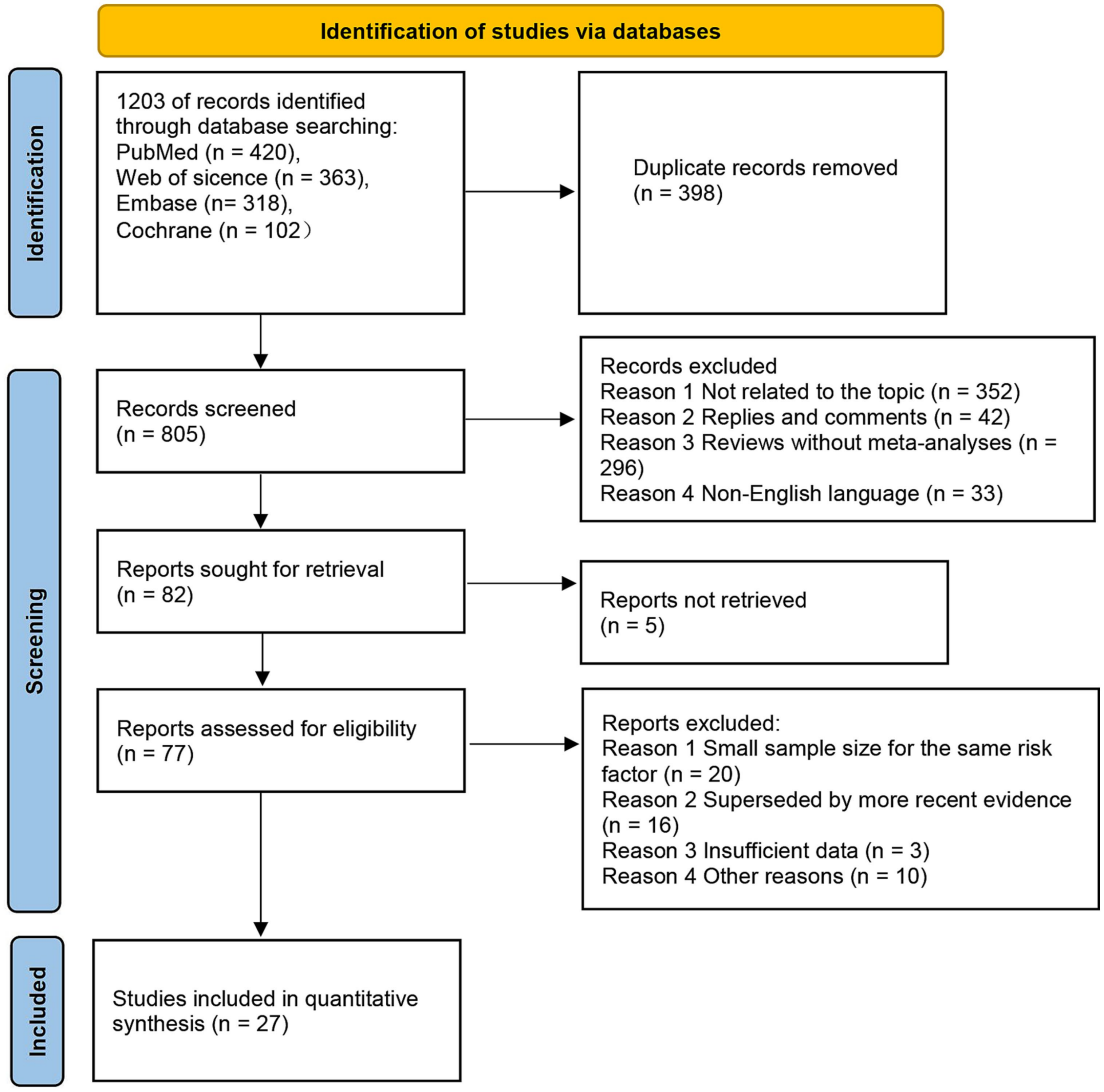
PRISMA flow diagram illustrating the study selection process, including identification, screening, eligibility assessment, and inclusion of meta-analyses.

All retrieved records were managed and screened using EndNote X9. Following duplicate removal, titles and abstracts were independently evaluated by two reviewers, with subsequent full-text review conducted to determine eligibility of meta-analyses. Any differences between reviewers were addressed by consulting a third reviewer. Reference citations within the included publications were independently examined to identify relevant studies that may not have been identified through the electronic search strategy (Figure 1).

### 2.9 Assessment of methodological quality

The methodological quality of included meta-analyses was independently evaluated by two investigators with AMSTAR, a well-validated and rigorous instrument for appraising systematic reviews and meta-analyses. Concurrently, evidence certainty for each health outcome was systematically classified as “high,” “moderate,” “low” or “very low” quality by employing the GRADE framework, thereby establishing evidentiary foundations for conclusive interpretations.

### 2.10 Data extraction

Two reviewers independently collected data from all eligible studies, including: (1) first author, (2) publication year, (3) investigated risk factors, (4) study population, (5) number of included studies, (6) number of cases and total participants, (7) study design (randomized controlled trial, cross-sectional, case–control, or cohort), (8) follow-up duration, (9) reported outcomes, and (10) effect estimates (RR, OR, or HR) with 95% confidence intervals. We additionally collected details on the meta-analytic model (random- or fixed-effects), heterogeneity measures (I^2^ and Cochran’s Q test), and small-study effect assessments (Egger’s test, Begg’s test, and funnel plots). When dose–response or subgroup analyses were conducted, *p* values were retrieved for nonlinearity and subgroup-specific estimates. Discrepancies between reviewers were resolved by a third reviewer.

### 2.11 Data summary

We extracted RR, OR, and HR with corresponding 95% CI, and recorded heterogeneity measures (I^2^ statistics and Cochran’s Q test) when available. Small-study effects were examined using Egger’s and Begg’s tests for systematic reviews and meta-analyses that included at least 11 studies. In the case of risk factors backed by high or moderate levels of evidence, sensitivity analyses were performed—where data allowed—to assess how each included study affected the overall robustness of the results. Dose–response analyses for AMD risk factors were also retrieved from the included meta-analyses. When the latest meta-analysis excluded certain clinical studies present in other analyses, we integrated data from those studies and performed a re-analysis. Statistical significance for heterogeneity was defined as *p* < 0.10, whereas all other tests used a threshold of *p* < 0.05. Review Manager (version 5.4) was used for evidence synthesis, while Stata version 15.1 was employed to conduct Egger’s and Begg’s tests as well as sensitivity analyses.

## 3 Results

### 3.1 Evidence synthesis and quality assessment

The process of literature search and study selection is summarized in the flow diagram shown in Figure 1. Following a systematic literature search, we identified and screened 805 non-duplicate studies. Through rigorous application of the predefined inclusion criteria, 27 eligible meta-analyses were included (9–19, 23–38). The meta-analytical synthesis identified 53 distinct risk factors, including 30 risk factors with significant associations and 23 with non-significant associations (Table 1). Eighteen detrimental associations and twelve beneficial associations demonstrated statistical significance. According to the GRADE evidence evaluation criteria, most results were rated as low or very low-quality evidence. In this comprehensive review, only three risk factors—blood lutein/zeaxanthin levels, fish consumption, and physical activity—were supported by moderate-quality evidence ratings. Personal characteristics and circulating biomarkers accounted for the largest proportion of the identified AMD-associated factors in this review.

**Table 1:** Risk factors for age-related macular degeneration.

Risk factors	Assessed with	Outcomes	Included MA	Sample size case/total	Effect measure	Estimates [95%CI]	No. of studies T/R/C/P	Effects model	I^2^; Q test *p* value	Egger test *p* value	AMSTAR	GRADE
Significant												
C-reactive protein levels	Highest vs. lowest	AMD	Chen Feng 2020 (24)	10,246/49106	OR	1.44 [1.31 to 1.60]	49/0/3/46	Random	84%; *p* < 0.0001	NA	8	Very low
Dietary omega-3 polyunsaturated fatty acids intake	Highest vs. lowest	Early AMD	Hong Jiang 2021 (18)	NA	RR	0.86 [0.77 to 0.96]	6/0/3/3	Random	0%;0.546	*P* > 0.05	9	Low
Dietary omega-3 polyunsaturated fatty acids intake	Highest vs. lowest	Late AMD	Hong Jiang 2021 (18)	NA	RR	0.71 [0.55 to 0.91]	9/1/1/7	Random	52.3%;0.033	*P* > 0.05	9	Very low
Cataract surgery	With vs. without	Late AMD	Lihong Yang 2022 (26)	NA	OR	1.80 [1.26 to 2.56]	5/0/0/5	Random	27.9%; 0.236	0.64	9	Low
Cataract surgery	With vs. without	The progression of AMD	Lihong Yang 2022 (26)	NA	OR	1.40 [1.10 to 1.80]	5/2/0/3	Random	2.2%; 0.394	NA	9	Low
Dementia/AD	With vs. without	AMD	Shi Song Rong 2019 (15)	NA/56276	OR	1.33 [1.12 to 1.57]	3/0/0/3	Random	0%; 0.52	*P* > 0.05	8	Very low
Mediterranean diet	With vs. without	AMD	Maria Angelia 2024 (27)	NA/26343	HR	0.82 [0.75 to 0.90]	8/0/5/3	Random	67%; 0.0002	NA	6	Low
Oral metformin use	With vs. without	AMD	Kai-Hsiang Liang 2022 (28)	NA/1446284	OR	0.81 [0.70 to 0.93]	9/0/5/4	Random	96%; *p* < 0.00001	NA	9	Very low
BMI	Per 1 kg/m^2^ increase in BMI	AMD	Zhang, Q. Y. 2016 (29)	1613/31151	RR	1.02 [1.01 to 1.04]	7/0/7/0	Random	58.6%; 0.025	NA	8	Low
Hypertension	With vs. without	AMD	Parsa Panahi 2023 (12)	NA/985	OR	1.512 [1.119 to 2.044]	3/0/0/3	Random	28.6%; 0.246	0.573	8	Low
Total cholesterol (TC)	Per 1 mmol/L increment of TC level	AMD	Wang, Y. 2016 (30)	NA/54862	RR	0.96 [0.93 to 0.99]	18/0/4/14	Random	58.9%; 0.001	*P* > 0.05	8	Very low
High-density lipoprotein cholesterol (HDL-C)	Per 1 mmol/L increment of HDL-C level	AMD	Wang, Y. 2016 (30)	NA/53981	RR	1.18 [1.01 to 1.35]	15/0/4/11	Random	53.8%; 0.007	*P* > 0.1	8	Very low
Low-density lipoprotein cholesterol (LDL-C)	Per 1 mmol/L increment of LDL-C level	AMD	Wang, Y. 2016 (30)	NA/27668	RR	0.93 [0.88 to 0.99]	10/0/1/9	Random	0%; 0.83	0.45	8	Very low
Triglycerides (TG)	Per 1 mmol/L increment of TG level	AMD	Wang, Y. 2016 (30)	NA/38467	RR	0.91 [0.87 to 0.94]	9/0/1/8	Random	2.6%; 0.42	*P* > 0.05	8	Low
Thyroid disease	With vs. without	AMD	Ziming Xu 2021 (14)	NA/61993	RR	1.25 [1.02 to 1.54]	7/0/2/5	Random	80.1%; *p* < 0.001	0.889	8	Very low
Ambient air pollution PM2.5	With vs. without	AMD	Zhuo Han 2024 (16)	NA/146051	OR	2.24 [1.25 to 4.00]	2/0/0/2	Random	0%; 0.536	*P* < 0.05	8	Very low
Ambient nitrogen dioxide (NO_2_)	With vs. without	AMD	Wu, J. 2024 (17)	NA/170888	OR	1.17 [1.09 to 1.25]	3/0/1/2	Random	96%; *P* < 0.00001	NA	*7*	Very low
Ambient ozone (O_3_)	With vs. without	AMD	Wu, J. 2024 (17)	NA/10523809	OR	1.06 [1.05 to 1.07]	3/0/0/3	Random	100%; *P* < 0.00001	NA	*7*	Very low
Chronic kidney disease	With vs. without	AMD	Yi-Ju Chen 2018 (13)	NA/335601	OR	1.35 [1.05 to 1.73]	12/0/3/9	Random	87.2%; *p* = 0.000	*P* < 0.05	8	Very low
Age	Younger vs. older	AMD	Raghad Babaker 2025 (9)	NA/22971	OR	1.11 [1.06 to 1.15]	11/0/5/6	Random	84%; P < 0.00001	NA	9	Very low
Gender	Male vs. Female	AMD	Raghad Babaker 2025 (9)	NA/18074	OR	1.63 [1.13 to 2.35]	7/0/2/5	Random	36%; 0.15	NA	9	Low
Diabetes	With vs. without	AMD	Raghad Babaker 2025 (9)	NA/8491	OR	1.44 [1.30 to 1.60]	6/0/3/3	Random	0%; 0.46	NA	9	Low
Cardiovascular diseases	With vs. without	AMD	Raghad Babaker 2025 (9)	NA/14058	OR	1.44 [1.11 to 1.87]	8/0/4/4	Random	67%; 0.003	NA	9	Very low
Physical activity	Sedentary vs. active	Early AMD	McGuinness, M. B. 2017 (35)	NA/38112	OR	0.92 [0.86 to 0.98]	8/0/3/5	Random	4.8%; 0.46	NA	8	Very low
Physical activity	Sedentary vs. active	Late AMD	McGuinness, M. B. 2017 (35)	NA/28854	OR	0.59 [0.49 to 0.72]	7/0/3/4	Random	23.5%; 0.46	NA	8	Moderate
Periodontal disease	With vs. without	AMD	Xuewen Lv 2020 (37)	5005/112240	RR	1.35 [1.07 to 1.70]	5/0/1/4	Random	80.4%; P < 0.0001	0.509	9	Very low
Smoking	With vs. without	AMD	Asiamah, R. 2025 (10)	NA	OR	7.61 [5.44 to 10.64]	4/0/0/4	Random	69.4%; 0.0033	NA	8	Very low
Alcohol consumption	Non/occasional vs. moderate (12–24 g/day)	Early AMD	Jingjing Zhang 2021 (11)	NA	OR	1.19 [1.03 to 1.37]	6/0/5/1	Random	0%; 0.487	NA	8	Very low
Alcohol consumption	Non/occasional vs. heavy (≥ 24 g/day)	Early AMD	Jingjing Zhang 2021 (11)	NA	OR	1.24 [1.10 to 1.39]	5/0/5/0	Random	38.8%; 0.162	NA	8	Low
Fish consumption	With vs. without	AMD	Wei Zhu 2016 (38)	4202/128988	RR	0.76 [0.65 to 0.90]	8/0/8/0	Random	49.6%; 0.053	0.068	8	Moderate
Blood lutein/zeaxanthin level	Highest vs. lowest	AMD	Jiang, H. 2022 (19)	NA	OR	0.53 [0.40 to 0.72]	9/0/1/8	Random	43.3%; 0.079	*P* > 0.05	9	Moderate
Blood β-carotene concentrations level	Highest vs. lowest	AMD	Jiang, H. 2022 (19)	NA	OR	0.48 [0.28 to 0.84]	6/0/1/5	Random	71.7%; 0.003	*P* > 0.05	9	Very low
Blood lycopene level	Highest vs. lowest	AMD	Jiang, H. 2022 (19)	NA	OR	0.70 [0.54 to 0.90]	6/0/1/5	Random	0%; 0.674	*P* > 0.05	9	Very low
Blood α-tocopherol level	Highest vs. lowest	AMD	Jiang, H. 2022 (19)	NA	OR	0.50 [0.31 to 0.81]	5/0/0/5	Random	34.4%; 0.192	*P* > 0.05	9	Low
Non-significant												
Obstructive sleep apnea (OSA)	With vs. without	AMD	Gabriella Bulloch 2024 (23)	77,058/3177362	OR	0.92 [0.24 to 3.58]	2/0/2/0	Random	100%; NA	NA	8	Very low
Sunlight exposure	With vs. without	AMD	Hongjie Zhou 2018 (31)	NA/43934	OR	1.10 [0.98 to 1.23]	14/0/1/13	Random	71.9%; <0.0001	0.25	8	Very low
Vegetables consumption	Highest vs. lowest	AMD	Monica Dinu 2019 (32)	4266/133904	RR	0.92 [0.82 to 1.03]	4/0/4/0	Random	13%; *p* = 0.15	NA	8	Very low
Fruits consumption	Highest vs. lowest	AMD	Monica Dinu 2019 (32)	4140/132525	RR	0.91 [0.82 to 1.01]	3/0/3/0	Random	19%; *p* = 0.29	NA	8	Very low
Grain consumption	Highest vs. lowest	AMD	Monica Dinu 2019 (32)	952/4335	RR	0.81 [0.64 to 1.02]	2/0/2/0	Random	58%; *p* = 0.07	NA	8	Very low
Nuts consumption	Highest vs. lowest	AMD	Monica Dinu 2019 (32)	1359/4711	RR	0.84 [0.62 to 1.13]	3/0/3/0	Random	52%; p = 0.15	NA	8	Very low
Meat consumption	Highest vs. lowest	AMD	Monica Dinu 2019 (32)	6771/101011	RR	1.11 [0.96 to 1.27]	6/0/6/0	Random	65%; *p* = 0.16	NA	8	Very low
Dairy products consumption	Highest vs. lowest	AMD	Monica Dinu 2019 (32)	970/73772	RR	1.07 [0.68 to 1.70]	3/0/3/0	Random	77%; *p* = 0.004	NA	8	Very low
Oils consumption	Highest vs. lowest	AMD	Monica Dinu 2019 (32)	2324/77078	RR	1.10 [0.98 to 1.23]	2/0/2/0	Random	1%; *p* = 0.36	NA	8	Very low
Butter consumption	Highest vs. lowest	AMD	Monica Dinu 2019 (32)	1909/7862	RR	1.04 [0.93 to 1.16]	2/0/2/0	Random	0%; *p* = 0.52	NA	8	Very low
Margarine consumption	Highest vs. lowest	AMD	Monica Dinu 2019 (32)	2346/79336	RR	1.05 [0.91 to 1.21]	3/0/3/0	Random	7%; p = 0.36	NA	8	Very low
Long sleep duration	>8 h vs. 7–8 h	AMD	Miao Zhou 2023 (33)	560/1168	OR	1.29 [0.71 to 2.33]	2/0/0/2	Random	0%; *p* = 0.859	*P* > 0.05	9	Very low
Short sleep duration	<7 h vs. 7–8 h	AMD	Miao Zhou 2023 (33)	560/1168	OR	1.49 [0.40 to 5.59]	2/0/0/2	Random	82.2%; *p* = 0.018	P > 0.05	9	Very low
Statin use	With vs. without	AMD	Memarzadeh, E. 2022 (34)	313,702/2063195	OR	0.93 [0.83 to 1.05]	22/0/11/11	Random	81.0%; ≤0.001	NA	8	Very low
Serum vitamin D levels	Per 10-ng/mL increase in serum vitamin D levels	AMD	Wu, W. 2016 (36)	NA	OR	0.91 [0.69 to 1.22]	8/0/2/6	Random	79.7%; <0.01	NA	8	Very low
Aspirin usage	With vs. without	AMD	Ruijia Yan 2022 (25)	NA/1002092	RR	1.108 [0.886 to 1.385]	16/2/6/8	Random	21%; NA	*P* > 0.05	8	Very low
Blood β-cryptoxanthin level	Highest vs. lowest	AMD	Jiang, H. 2022 (19)	NA	OR	0.48 [0.23 to 1.00]	6/0/1/5	Random	83.5%; 0.000	*P* > 0.05	9	Very low
Blood retinol level	Highest vs. lowest	AMD	Jiang, H. 2022 (19)	NA	OR	0.61 [0.12 to 3.16]	3/0/0/3	Random	70.7%; 0.033	*P* > 0.05	9	Very low
Thyroid medication	With vs. without	AMD	Ziming Xu 2021 (14)	NA/118420	RR	1.26 [0.92 to 1.72]	7/0/3/4	Random	69.0%; p = 0.004	0.226	8	Very low
Overweight	Overweight (25–29.9 kg/m^2^) vs. Normal weight (18.5–25 kg/m^2^)	AMD	Zhang, Q. Y. 2016 (29)	NA/28086	RR	1.03 [0.90 to 1.17]	5/0/5/0	Random	23.0%; 0.268	0.64	8	Very low
Obesity	Obesity (≥30 kg/m^2^) vs. Normal weight (18.5–25 kg/m^2^)	AMD	Zhang, Q. Y. 2016 (29)	NA/31151	RR	1.07 [0.94 to 1.21]	7/0/7/0	Random	58.6%; 0.025	0.80	8	Very low
Underweight	Underweight (<18.5 kg/m^2^) vs. Normal weight (18.5–25 kg/m^2^)	AMD	Zhang, Q. Y. 2016 (29)	NA/27652	RR	0.96 [0.74 to 1.18]	4/0/4/0	Random	0%; 0.807	0.69	8	Very low
Cerebrovascular diseases	With vs. without	AMD	Raghad Babaker 2025 (9)	NA/7120	OR	1.66 [0.73 to 3.79]	3/0/1/2	Random	0%; 0.74	NA	9	Very low

OR, odds ratio; RR, relative risk; HR, hazard ratio; OSA, Obstructive Sleep Apnea; AD, Alzheimer’s disease; TC, Total Cholesterol; HDL-C, High-density Lipoprotein Cholesterol; LDL-C, Low-density Lipoprotein Cholesterol; TG, Triglycerides; NO2, Ambient Nitrogen Dioxide; O3, Ambient Ozone; CKD, Chronic Kidney Disease; CI, confidence interval; T, total No. of studies; R, randomized controlled trials; C, cohort studies; P, population-based case–control and/or cross-sectional studies; AMSTAR, a measurement tool to assess systematic reviews; GRADE, Grading of Recommendations Assessment, Development, and Evaluation; NA, not available.

### 3.2 Lifestyle

Fish consumption demonstrated a significant protective effect against AMD (RR: 0.76, 95% CI: 0.65 to 0.90, GRADE: moderate) (38). Additionally, a potential dose–response relationship was evaluated, showing that fish consumption once per week was associated with an 11% lower risk of AMD (RR: 0.89, 95% CI: 0.83 to 0.96) (38). Individuals with higher adherence to the Mediterranean diet (MD) exhibited a reduced risk of developing AMD (HR: 0.82; 95% CI: 0.75 to 0.90, GRADE: low) (27). A meta-analysis of 21 studies indicated that higher dietary omega-3 PUFA intake was significantly associated with a 14% decreased risk of early AMD (RR: 0.86, 95% CI: 0.77 to 0.96, GRADE: low) and a 29% decreased risk of late AMD (RR: 0.71, 95% CI: 0.55 to 0.91, GRADE: very low) (18), with each 1 g/day increment in omega-3 PUFA consumption linked to 6% (RR: 0.94, 95% CI: 0.90 to 0.98) and 22% (RR: 0.78, 95% CI: 0.65 to 0.94) decreased risks for early and late AMD (18).

Results of a meta-analysis suggest that increased physical activity is linked to a significantly reduced risk of both late AMD (OR: 0.59; 95% CI: 0.49 to 0.72, GRADE: moderate) and early AMD (OR: 0.92; 95% CI: 0.86 to 0.98, GRADE: very low) relative to sedentary behavior (35). Smoking is a significant risk factor for AMD, demonstrating a strong association with AMD (OR: 7.61, 95% CI: 5.44 to 10.64, GRADE: very low) (10). Similarly, moderate (12–24 g/day; OR: 1.19, 95% CI: 1.03 to 1.37, GRADE: very low) and heavy (≥ 24 g/day; OR: 1.24, 95% CI: 1.10 to 1.39, GRADE: low) alcohol consumption exhibited an elevated risk of early AMD compared to nondrinkers or occasional drinkers (11).

**Figure 2:**
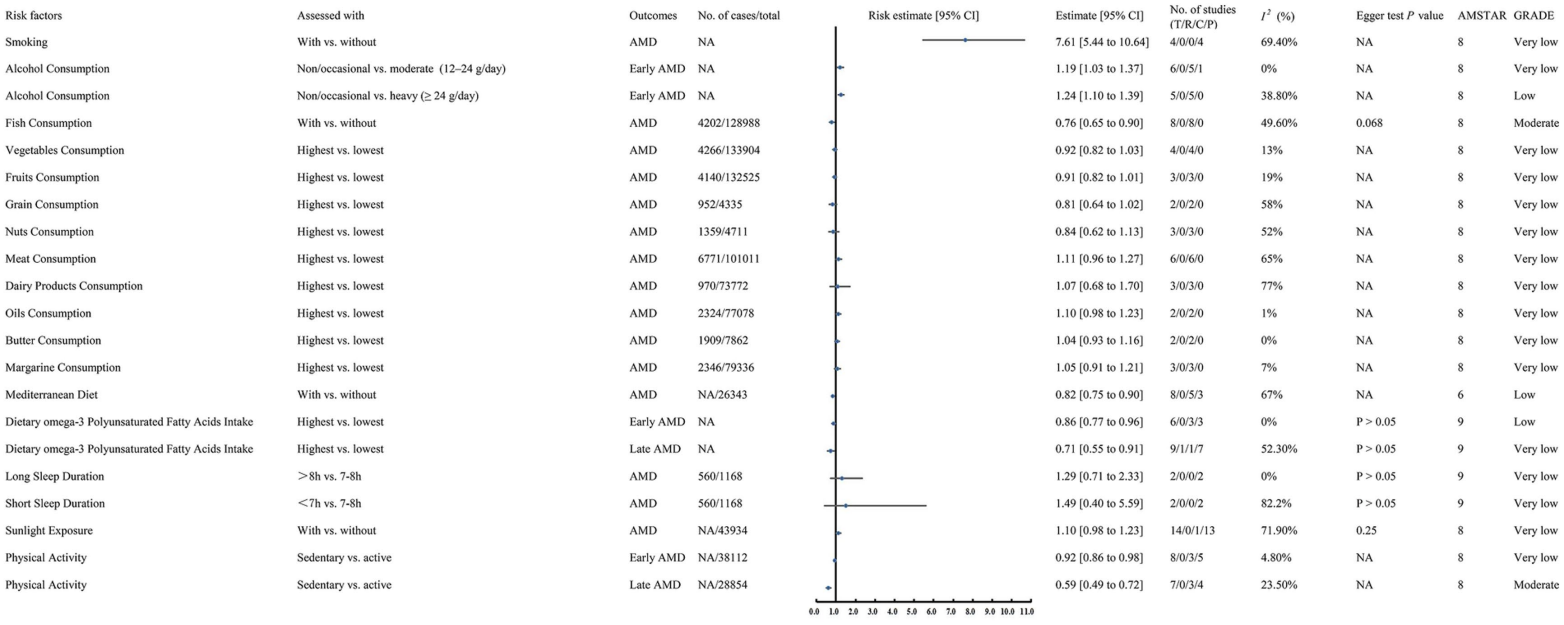
Forest plot of the impact of lifestyle-related risk factors and AMD risk.

A meta-analysis revealed nonsignificant associations between AMD incidence and intake of vegetables (RR: 0.92, 95% CI: 0.82 to 1.03, GRADE: very low), fruits (RR: 0.91, 95% CI: 0.82 to 1.01, GRADE: very low), grains (RR: 0.81, 95% CI: 0.64 to 1.02, GRADE: very low), nuts (RR: 0.84, 95% CI: 0.62 to 1.13, GRADE: very low), meat (RR: 1.11, 95% CI: 0.96 to 1.27, GRADE: very low), dairy products (RR: 1.07, 95% CI: 0.68 to 1.70, GRADE: very low), or oils (RR: 1.10, 95% CI: 0.98 to 1.23, GRADE: very low), butter (RR: 1.04, 95% CI: 0.93 to 1.16, GRADE: very low), margarine (RR: 1.05, 95% CI: 0.91 to 1.21, GRADE: very low) (32). Furthermore, neither long sleep duration (>8 h/day; OR: 1.29, 95% CI: 0.71 to 2.33, GRADE: very low) (33), short sleep duration (<7 h/day; OR: 1.49, 95% CI: 0.40 to 5.59, GRADE: very low) (33), nor sunlight exposure (OR: 1.10, 95% CI: 0.98 to 1.23, GRADE: very low) demonstrated significant associations with AMD risk (31) (Figure 2).

### 3.3 Personal characteristics and circulating biomarkers

Higher blood lutein/zeaxanthin levels were linked to a reduced risk of AMD (OR: 0.53, 95% CI: 0.40 to 0.72, GRADE: moderate) (19). Notably, comparable inverse associations were identified for *α*-tocopherol (OR: 0.50, 95% CI: 0.31 to 0.81, GRADE: low), *β*-carotene (OR: 0.48, 95% CI: 0.28 to 0.84, GRADE: very low), and lycopene (OR: 0.70, 95% CI: 0.54 to 0.90, GRADE: very low) (19). Contrarily, no significant associations were detected between AMD risk and blood β-cryptoxanthin levels (OR: 0.48, 95% CI: 0.23 to 1.00, GRADE: very low), blood retinol concentrations (OR: 0.61, 95% CI: 0.12 to 3.16, GRADE: very low) (19), or serum vitamin D levels (OR: 0.91, 95% CI: 0.69 to 1.22, GRADE: very low) (36).

A meta-analysis synthesizing 11 studies demonstrated a statistically significant elevation in AMD incidence in older versus younger adults (OR: 1.11, 95% CI: 1.06 to 1.15, GRADE: very low) (9). Within the same meta-analysis, analysis of eight studies showed significantly higher AMD incidence in males than females (OR: 1.63, 95% CI: 1.13 to 2.35, GRADE: low) (9).

Lipid metabolism contributes to the pathogenic mechanism of AMD, each 1 mmol/L increment in total cholesterol (TC; RR: 0.96, 95% CI: 0.93 to 0.99, GRADE: very low), low-density lipoprotein cholesterol (LDL-C; RR: 0.93, 95% CI: 0.88 to 0.99, GRADE: very low), and triglycerides (TG; RR: 0.91, 95% CI: 0.87 to 0.94, GRADE: low) was significantly linked to a lower risk of AMD (30). Conversely, high-density lipoprotein cholesterol (HDL-C; RR: 1.18, 95% CI: 1.01 to 1.35, GRADE: very low) increment was linked to an elevated AMD risk (30). Separately, another study demonstrated no significant associations between AMD and individuals across different BMI categories: underweight (RR: 0.96, 95% CI: 0.74 to 1.18 GRADE: very low), overweight (RR: 1.03, 95% CI: 0.90 to 1.17 GRADE: very low), or obesity (RR: 1.07, 95% CI: 0.94 to 1.21 GRADE: very low) (29). Higher BMI was linearly associated with increased AMD risk, with each 1 kg/m^2^ rise within the overweight and obese categories corresponding to a 2% increase in risk (RR: 1.02, 95% CI: 1.01–1.04; GRADE: low) (29).

**Figure 3:**
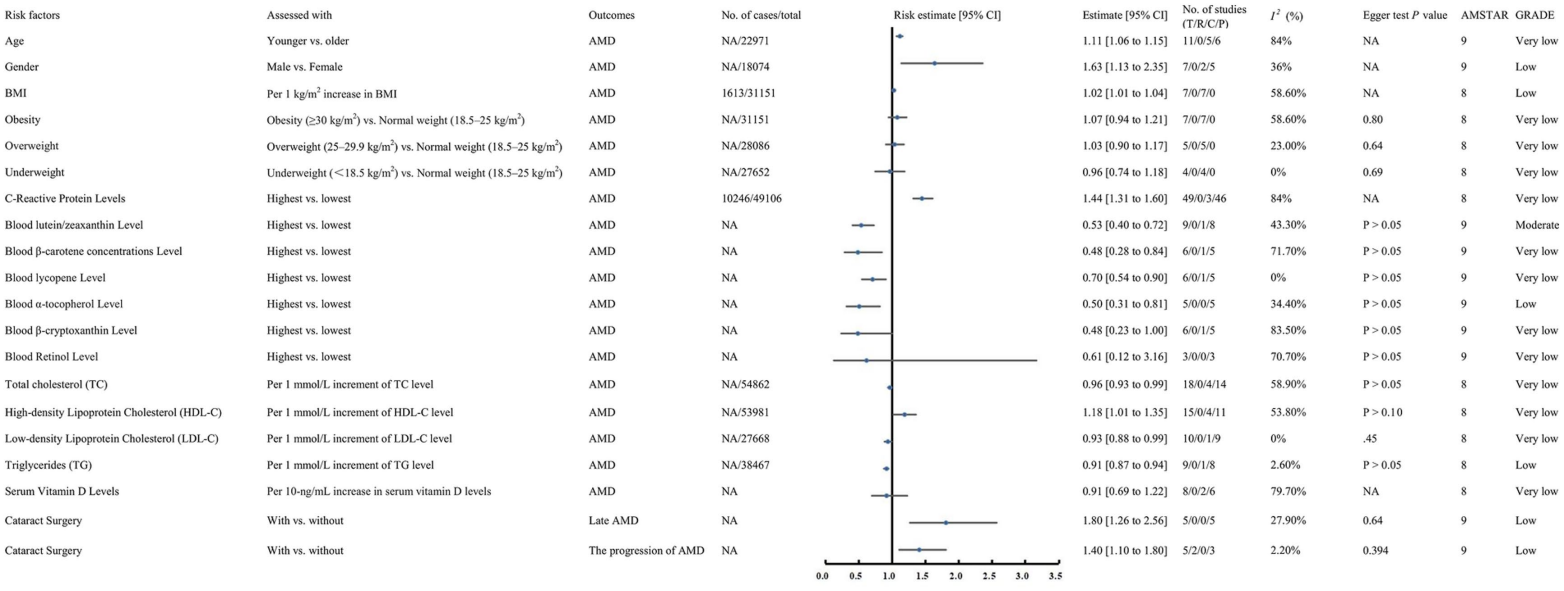
Forest plot of the impact of personal characteristics and circulating biomarkers on AMD risk.

Given AMD’s inflammatory links, this study revealed that AMD patients exhibited markedly elevated CRP levels (OR: 1.44, 95% CI: 1.31 to 1.60, GRADE: very low) (24). History of cataract surgery showed a significant association with an increased incidence of late AMD (OR: 1.80, 95% CI: 1.26 to 2.56, GRADE: low) and progression of AMD (OR: 1.40, 95% CI: 1.10 to 1.80, GRADE: low) (26) (Figure 3).

### 3.4 Underlying diseases

**Figure 4:**
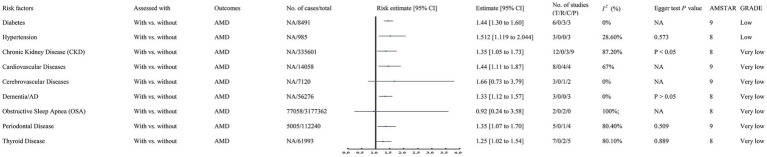
Forest plot of the impact of underlying diseases on AMD risk.

As common systemic diseases, patients with hypertension (OR: 1.512, 95% CI: 1.119 to 2.044, GRADE: low) and diabetes (OR: 1.44, 95% CI: 1.30 to 1.60, GRADE: low) exhibit an elevated risk of AMD development (9, 12). Similarly, a meta-analysis demonstrated a significant positive association between chronic kidney disease (CKD) and AMD (OR: 1.35, 95% CI: 1.05 to 1.73, GRADE: very low) (13). Among older populations, dementia—including Alzheimer’s disease (AD)—is a leading cause of disability and dependency. Evidence from a large meta-analysis indicated that dementia/AD is significantly linked to higher AMD risk (OR: 1.33, 95% CI: 1.12 to 1.57, GRADE: very low) (15). Periodontal disease (PD), an inflammatory and destructive disease, was also associated with a higher incidence of AMD compared with non-PD individuals (RR: 1.35, 95% CI: 1.07 to 1.70, GRADE: very low) (37). Furthermore, thyroid disease was linked to a 25% higher risk of AMD (RR: 1.25; 95% CI: 1.02 to 1.54, GRADE: very low) (14). Cardiovascular diseases, as prevalent conditions in aging populations, were also associated with higher AMD risk (OR: 1.44, 95% CI: 1.11 to 1.87, GRADE: very low) (9). Conversely, cerebrovascular diseases (OR: 1.66, 95% CI: 0.73 to 3.79, GRADE: very low) and obstructive sleep apnea (OSA; OR: 0.92, 95% CI: 0.24 to 3.58, GRADE: very low) show no significant AMD associations (9, 23) (Figure 4).

### 3.5 Pharmacological exposures

**Figure 5:**
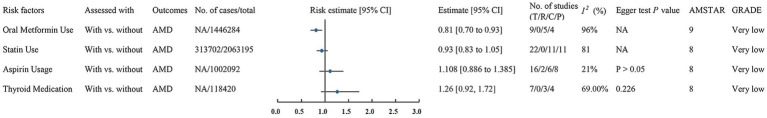
Forest plot of the impact of pharmacological exposures on AMD risk.

Oral metformin use was associated with reduced AMD risk (OR: 0.81, 95% CI: 0.70 to 0.93, GRADE: very low) (28). In contrast, no significant associations were detected for statin use (OR: 0.93, 95% CI: 0.83 to 1.05, GRADE: very low) (34), aspirin use (RR: 1.108, 95% CI: 0.886 to 1.385, GRADE: very low) (25), or thyroid medication (RR: 1.26, 95% CI: 0.92 to 1.72, GRADE: very low) in relation to AMD incidence (14) (Figure 5).

### 3.6 Environmental factors

**Figure 6:**
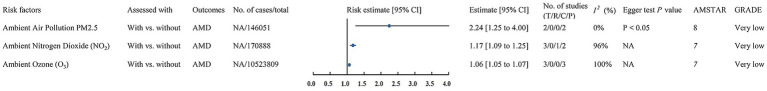
Forest plot of the impact of environmental factors on AMD risk.

Exposure to ambient air pollution was reported to be associated with higher AMD risk, including nitrogen dioxide (NO_2_; OR: 1.17, 95% CI: 1.09 to 1.25, GRADE: very low) (17), ozone (O_3_; OR: 1.06, 95% CI: 1.05 to 1.07, GRADE: very low) (17), and PM_2.5_ (OR: 2.24, 95% CI: 1.25 to 4.00, GRADE: very low) (16) (Figure 6).

### 3.7 Heterogeneity

We extracted heterogeneity measures for all risk factors. Approximately 49.1% of the risk factors showed high heterogeneity (*I*^2^ > 50% or *p* value of Cochran’s *Q*-test < 0.1). Among these heterogeneous factors, smoking, age, MD, BMI, TC, HDL-C, C-reactive protein levels, dietary omega-3 polyunsaturated fatty acids intake, oral metformin use, thyroid disease, NO_2_, O_3_, chronic kidney disease, cardiovascular diseases, periodontal disease, blood *β*-carotene concentration were significantly associated with AMD incidence. Therefore, these statistically significant findings should be interpreted with caution, as the effect sizes may vary across populations and study settings. Conversely, the remaining 50.9% of risk factors exhibited low heterogeneity.

### 3.8 Assessment of risk of bias

Publication bias was assessed using Egger’s test extracted from individual studies, which detected evidence of bias for three risk factors: ambient air pollution PM_2.5_, chronic kidney disease, and fish consumption. While 43.9% of studies showed no significant publication bias, the remainder either did not assess publication bias or had too few studies for valid assessment.

### 3.9 AMSTAR and GRADE

Across all risk factors, the median AMSTAR score was 8 (range 6–9) (Table 1), and Supplementary Table S2 provides the corresponding scores for each outcome. In terms of GRADE, physical activity, fish consumption, and blood lutein/zeaxanthin levels were graded as moderate-quality evidence. Dietary omega-3 polyunsaturated fatty acids intake, cataract surgery, MD, BMI, hypertension, TG, gender, diabetes, alcohol consumption, and blood *α*-tocopherol levels were classified as low-quality evidence because of indirectness, inconsistency, plausible confounding, publication bias, or imprecision. The remaining risk factors were classified as very low-quality evidence due to risk of bias, indirectness, inconsistency, plausible confounding, publication bias, or imprecision. The detailed GRADE assessment results are provided in Supplementary Table S3. Sensitivity analyses of outcomes rated as moderate quality showed no changes in either the direction or significance of the associations.

## 4 Discussion

### 4.1 Principal findings and possible explanations

AMD is a progressive degenerative disease of the macula and a key factor in vision loss worldwide. As a complex and multifactorial condition, AMD involves both modifiable and non-modifiable risk factors (39). The burden of AMD worldwide is expected to grow significantly in the coming decades. For this reason, understanding its risk factors is important for clarifying disease mechanisms and improving early identification and prevention (39).

This umbrella review examined the associations between AMD and multiple risk factors. It included data from 27 meta-analyses and identified 53 distinct risk factors. Of these, 30 factors showed statistically significant associations. They were distributed across five domains: personal characteristics and circulating biomarkers, environmental factors, lifestyle, underlying diseases, and pharmacological exposures. Among the significant associations, 18 factors were associated with increased AMD risk, whereas 12 were associated with reduced risk. The reported effect sizes varied across studies, with odds ratios ranging from 0.48 to 7.61.

This review integrated evidence from multiple meta-analyses and assessed methodological quality and certainty using AMSTAR and GRADE. However, the overall certainty of the evidence remained limited. Most associations were supported by low or very low-quality evidence. Three risk factors were supported by moderate-quality evidence: physical activity, fish consumption, and blood lutein/zeaxanthin levels. Many studies showed considerable statistical heterogeneity, and I^2^ values exceeded 50% in several meta-analyses. This variability may be related to differences in population characteristics, exposure definitions, and outcome assessment. For this reason, statistically significant pooled estimates should not be interpreted solely on the basis of their *p* values. For associations with substantial heterogeneity, the direction of association may be informative, but the exact effect size may be less stable across populations and study settings. These findings should therefore be regarded as suggestive rather than definitive, particularly when the certainty of evidence was also rated as low or very low. AMD is a multifactorial disease, and lifestyle and systemic factors may contribute substantially to its development. The following sections focus on these modifiable factors and their potential roles in AMD onset and progression.

### 4.2 Lifestyle factors are key determinants influencing the risk of AMD

Lifestyle factors are important modifiable contributors to AMD risk. Smoking is widely recognized as a key modifiable risk factor for AMD. This umbrella review showed that both current and former smokers had a more than seven-fold higher risk of AMD compared with never-smokers (10). This association may be dose-dependent. Higher smoking intensity or longer duration was associated with greater AMD risk, and a dose–response relationship was reported (40). Smoking may affect AMD development through several mechanisms. Tobacco-derived free radicals and phagocyte-generated reactive oxygen species can accelerate lipid peroxidation, leading to RPE damage and impaired cellular homeostasis (40). It may also disrupt intracellular clearance pathways and promote the accumulation of toxic metabolic byproducts within the RPE (40). In addition, smoking-induced inflammation and complement activation may promote drusen formation and photoreceptor damage (7, 8). Smoking may further contribute to choroidal hypoxia and atherosclerosis, which can reduce blood supply to the outer retina (40). Individuals who quit smoking may have a lower likelihood of developing AMD. Although former smokers remain at a higher risk than never-smokers, studies suggest that after 20 years of cessation, this risk may become comparable to that of never-smokers (41). Clinicians should pay attention to smoking history and consider smoking cessation counseling as part of routine ophthalmic care (42).

Physical activity was associated with a lower risk of both early and late AMD. For late AMD, this association was supported by moderate-quality evidence (35). Regular physical activity may strengthen antioxidant defenses and reduce both retinal oxidative stress and chronic systemic inflammation (43). It may also improve vascular function, thereby enhancing oxygen and nutrient delivery to the outer retina and reducing hypoxic stress on the RPE (44). However, people who exercise regularly often maintain other healthy behaviors, such as better diet and lower smoking rates. This may partly confound the association and overestimate the independent effect of physical activity (35). Even so, physical activity still contributes importantly to AMD prevention through both ocular and systemic effects. Dietary habits also influence AMD risk. One meta-analysis showed that both moderate (12–24 g/day) and heavy (≥ 24 g/day) alcohol consumption were linked to an increased risk of early AMD (11, 38). Alcohol may increase reactive oxygen species (ROS) levels, reduce antioxidant capacity, and generate harmful radicals such as 1-hydroxylethyl radical (HER) (45). These changes may lead to oxidative damage in retinal tissue (45). The included meta-analysis evaluated alcohol consumption in relation to early AMD, but did not provide pooled evidence for late AMD. Therefore, no conclusion can be drawn regarding the association between alcohol consumption and late-stage disease based on the current umbrella review. However, experimental studies suggest that chronic alcohol exposure may promote angiogenesis and choroidal neovascularization (46). This indicates that a possible role of alcohol in late AMD cannot be excluded. These findings suggest that limiting excessive alcohol consumption may be reasonable in individuals at risk of AMD. Our review showed that omega-3 polyunsaturated fatty acids (PUFAs) were linked to a lower risk of both early and late AMD (18). Omega-3 PUFAs such as docosahexaenoic acid (DHA) and eicosapentaenoic acid (EPA) may help protect retinal function and reduce AMD risk (18). These long-chain fatty acids constitute essential components of photoreceptor membranes (18). They may help reduce retinal oxidative stress, inhibit VEGF-driven neovascularization, and protect RPE cells from apoptosis (18). Dietary sources of these omega-3 fatty acids are mainly fish, especially oily fish including salmon, tuna, and sardines (38). Higher fish consumption was related to a lower risk of AMD in our review, and this association was supported by moderate-quality evidence (38). This protective effect may be partly attributable to the omega-3 fatty acids found in fish. Beyond fish consumption alone, adherence to the MD has also been linked to a reduced risk of AMD (27). This dietary pattern includes fish, fruits, vegetables, legumes, whole grains, and olive oil (27). It can provide nutrients such as omega-3 fatty acids, lutein, zeaxanthin, polyphenols, and vitamins C and E (27). These nutrients may reduce oxidative stress and inflammation while supporting vascular function, which may contribute to a lower risk of AMD (27).

### 4.3 Personal characteristics and circulating biomarkers associated with AMD risk

Several individual characteristics and circulating biomarkers were associated with AMD risk, including both modifiable and non-modifiable factors. AMD risk increases markedly with age (39). Evidence suggests that the incidence of advanced AMD nearly doubles with each decade after the age of 60 (47). One study observed that the prevalence of early AMD was 3.5% in adults aged 55–59 years, increasing to 17.6% in the oldest age group (≥85 years), while late AMD prevalence rose from 0.1 to 9.8% across the same age range (48). Several mechanisms linked to aging may be involved in the initiation and advancement of AMD (49). Declines in phagocytic, autophagic, and mitochondrial function in RPE cells can lead to the accumulation of photoreceptor debris and impaired energy metabolism (50, 51). Age-associated thickening of Bruch’s membrane and lipid deposition may further impair nutrient transport and waste removal, thereby aggravating RPE dysfunction (52). Chronic low-grade inflammation and aberrant complement activation may contribute to immune-mediated tissue damage and the formation of pathological deposits (53). Age-associated choroidal thinning and reduced blood flow may also compromise oxygen supply to the macula (49). These changes can disrupt retinal homeostasis and contribute to AMD progression (49). In our review, male sex was linked to a higher risk of AMD (9). This differs from some earlier studies that observed a higher prevalence of AMD among women (9). This discrepancy may be partly attributed to women’s longer life expectancy, potentially leading to a higher proportion of older female patients in population-based studies (39). Some studies suggest that estrogens may favorably modulate serum lipid profiles and exert antioxidant effects (54). This may partly explain the lower AMD risk reported in women receiving hormone therapy (HT) (39, 54).

Carotenoids such as lutein, zeaxanthin, *β*-carotene, and lycopene, as well as antioxidant vitamins including *α*-tocopherol, β-cryptoxanthin, and retinol, may have protective effects against AMD (19). These micronutrients may help limit retinal damage through antioxidant and anti-inflammatory effects (55). Their protective effects may be achieved through quenching singlet oxygen, scavenging reactive oxygen species, preserving lysosomal integrity, and reducing RPE apoptosis (56, 57). Lutein and zeaxanthin can also absorb blue light, which may provide additional protection to the retina (58). These findings indicate that adequate intake of multiple nutrients may help lower the risk of AMD. By contrast, elevated high-density lipoprotein cholesterol (HDL-C) was associated with a higher risk of AMD (30). In contrast, higher triglyceride (TG) and low-density lipoprotein cholesterol (LDL-C) levels were inversely associated (30). This observation appears to challenge the conventional cardiovascular paradigm, which categorizes HDL-C as “good” cholesterol and LDL-C as “bad” cholesterol. This pattern suggests that lipid metabolism in the retina and choroid may differ from that in systemic circulation. In a cohort of 106,703 individuals, elevated plasma HDL-C and decreased LDL-C levels were linked to a higher risk of AMD (59). However, another meta-analysis found no significant differences in systemic TG, TC, LDL-C, or HDL-C levels between AMD patients and controls, regardless of disease stage (60). These inconsistent results indicate that the association between lipid profiles and AMD is still not fully understood. One possibility is that elevated HDL-C levels may contribute to the formation of drusen (61). The pathogenic role of HDL-C may also be related to functional impairment (62). Under conditions of oxidative stress and chronic inflammation, HDL may lose its normal biological activity and become pro-inflammatory (63). Genome-wide association studies have revealed multiple lipid-related genes, including LIPC, ABCA1, CETP, and LPL, that are associated with both HDL regulation and AMD susceptibility (64–66). These variants may influence HDL levels and function by affecting reverse cholesterol transport, hepatic lipase activity, and triglyceride transfer between lipoproteins (64–66). This finding suggests that the role of lipid metabolism in AMD may be more complex than previously thought. However, the findings for LDL-C, TG, and TC remain inconsistent, and further clarification is still needed.

Since elevated lipid levels are often found in individuals with higher body mass index (BMI), we further evaluated the relationship between BMI and AMD risk. A weak dose–response relationship was identified between higher BMI and a higher risk of AMD, with the association being more pronounced in late-stage disease (29). The exact mechanisms linking obesity to AMD remain incompletely understood, but several plausible pathways have been proposed. First, excess adiposity may impair the bioavailability of macular carotenoids including zeaxanthin and lutein, which are essential for retinal protection. In individuals with obesity, these fat-soluble carotenoids are more readily sequestered into adipose tissue, thereby reducing their availability for uptake by the macula (67, 68). Second, obesity is linked to increased levels of pro-inflammatory cytokines, including TNF-*α*, MCP-1, and CRP (69). These inflammatory mediators may impair RPE function and promote retinal degeneration (69). Third, obesity often coexists with other metabolic disturbances, such as hypertension (12), diabetes (9), and cardiovascular diseases (9), which are also associated with increased AMD risk.

C-reactive protein (CRP), traditionally considered a marker of systemic low-grade inflammation, may also be involved in AMD pathogenesis (24). Monomeric CRP (mCRP) is a conformationally altered form of CRP generated in the inflammatory microenvironment. Complement factor H (FH) binds to mCRP to attenuate its proinflammatory activity; however, in AMD patients carrying the CFH Y402H variant, FH exhibits impaired binding to mCRP, potentially allowing its proinflammatory effects to proceed unchecked (70). Experimental evidence also suggests that mCRP can upregulate IL-8 and CCL2, thereby promoting leukocyte recruitment and inflammatory signaling at the outer retina–choroid interface (71). Clinically, elevated systemic CRP has been associated with choroidal thinning in AMD, supporting a potential link between systemic inflammation, choroidal vulnerability, and AMD progression (72).

A history of cataract surgery was associated with late AMD and AMD progression in the included meta-analysis (26). This finding may have several explanations. First, cataract surgery can cause a short-term inflammatory response within the eye, and this postoperative inflammation may affect the RPE and choroid, which are central to AMD development (73). Oxidative stress may also increase after surgery and further contribute to retinal pigment epithelial damage (74, 75). The loss of the natural lens may also alter the amount and spectrum of light reaching the retina (76). Nevertheless, whether this change has a clinically meaningful effect on AMD progression remains uncertain, as evidence for a protective role of blue-light filtering intraocular lenses is still inconclusive (76). Beyond these biological mechanisms, detection bias should also be considered. Cataract can reduce the quality of macular imaging and may obscure subtle AMD changes before surgery (77). After cataract removal, clearer media and more frequent postoperative follow-up may increase the detection of pre-existing or mild AMD, leading to possible detection bias. Therefore, the observed association does not necessarily indicate that cataract surgery directly causes AMD. Further prospective studies with standardized preoperative retinal imaging and long-term follow-up are needed to clarify this relationship.

### 4.4 Impact of systemic diseases on AMD risk

The complex relationship between diabetes and AMD is partly due to the heterogeneous classifications and staging systems of both diseases, complicating direct correlations between their subtypes (78). Hyperglycemia may promote the development of AMD by promoting the accumulation of advanced glycation end-products (AGEs) and inducing hemodynamic disturbances through mechanisms such as oxidative stress, chronic inflammation, and mitochondrial dysfunction (79). Elevated glucose levels may also upregulate vascular endothelial growth factor (VEGF) expression, a central mediator in choroidal neovascularization (CNV) (79). Furthermore, diabetes and AMD share common risk factors, including aging, obesity, and unhealthy lifestyle behaviors (80). Similar to diabetes, hypertension has been associated with the development of AMD through overlapping mechanisms involving oxidative stress and chronic inflammation. It may also contribute to AMD progression through activation of the renin–angiotensin–aldosterone system (RAAS) (81). RAAS activation can foster a pro-inflammatory microenvironment that facilitates macrophage infiltration and angiogenesis (82). Angiotensin II (Ang II), a central effector of RAAS, can induce apoptosis of retinal endothelial cells, impair choroidal perfusion by constricting retinal vessels, and promote CNV by upregulating VEGFR-2 expression (82). Notably, Ang II has also been shown to increase the activity of matrix metalloproteinase-2 (MMP-2) and its key regulator MMP-14 in the RPE, leading to basement membrane degradation and potentially facilitating the accumulation of sub-RPE deposits (83). These findings suggest that effective control of blood pressure and blood glucose may help reduce AMD risk. Emerging evidence suggests that CKD may contribute to AMD pathogenesis through overlapping genetic and pathophysiological pathways (13). Both conditions share common susceptibility genes such as complement factor H and apolipoprotein E (13), as well as activation of the RAAS, which may exacerbate retinal inflammation (84). Impaired renal function leads to elevated oxidative stress due to reduced clearance of nitrogenous waste, which may accelerate lipid accumulation in Bruch’s membrane (85). Lipid deposition may promote degeneration and calcification of elastin and collagen fibers in Bruch’s membrane (85). As a result, the membrane may become more fragile and more susceptible to rupture under elevated vascular pressure, which may facilitate choroidal neovascularization (85).

Dementia/Alzheimer’s disease (AD) may be an independent risk factor for AMD (15). Both diseases involve chronic inflammation, complement activation, oxidative stress, and extracellular amyloid-*β* (Aβ) accumulation (86). Recent studies suggest that brain-derived Aβ can be transported and deposited in perivascular spaces adjacent to retinal vessels (87). This abnormal accumulation may trigger glial activation and retinal degeneration and may contribute to visual impairment in patients with AD (87). Genome-wide association studies (GWAS) have identified overlapping immune-related risk loci in AD and AMD, including APOE, ABCA1, and PILRA (86). However, the effects of these shared variants may differ across tissues. For example, the APOE ε4 allele increases the risk of AD but appears to be protective in AMD (88). These findings suggest that AD and AMD have some overlapping features. The possible association between periodontal disease (PD) and AMD has received increasing attention because they share some risk factors and inflammatory features (37). PD may promote systemic inflammation by allowing bacterial components such as endotoxins to enter the circulation (89). This may activate immune cells and trigger inflammatory responses in distant tissues (89).

Certain thyroid diseases have also been implicated in an elevated risk for AMD (14). A recent study reported that both hypothyroidism and hyperthyroidism demonstrated a significant association with a higher risk of exudative AMD, potentially through disruption of systemic oxidative balance, which renders the RPE more vulnerable to oxidative damage (90, 91). Notably, experimental inhibition of thyroid hormone (TH) signaling has been shown to protect RPE and photoreceptors in AMD models (92). Second, thyroid dysfunction, particularly hypothyroidism, is associated with atherogenic lipid profiles, which may promote cholesterol-rich drusen and Bruch’s membrane thickening (93–95). Additionally, TH can activate non-genomic signaling via integrin αvβ3, enhancing VEGF expression and angiogenesis, providing a plausible link to neovascular AMD (96). Additional well-designed longitudinal and mechanistic studies are required to substantiate these associations and elucidate how thyroid hormone imbalance influence AMD pathogenesis in older populations.

### 4.5 Pharmacological exposures and environmental pollutants in relation to AMD risk

Current evidence suggests that metformin use is linked to a reduced risk of developing AMD, with this protective effect observed in both diabetic and non-diabetic populations (28, 97). Mechanistically, metformin may confer this benefit by enhancing mitophagy in retinal pigment epithelium cells, facilitating the removal of damaged mitochondria, lowering mitochondrial reactive oxygen species generation, and mitigating chronic inflammatory responses that contribute to AMD pathogenesis (98). Recent preclinical animal studies show that intravitreal metformin inhibits choroidal neovascularization, reduces inflammatory cell infiltration, preserves retinal structure, and downregulates angiogenesis- and inflammation-related genes, supporting its potential as an accessible therapy for neovascular AMD (99). In animal experiments, oral metformin reduces AMD risk by reshaping the gut microbiome, increasing the abundance of Bifidobacterium and Akkermansia, and elevating fecal levels of butyrate, succinate, and cholic acid (100). In microbiome-depleted mice, fecal microbiota transplantation restored the anti-CNV effect of metformin (100). These findings suggest that metformin may have potential in AMD treatment. We also identified several environmental pollutants, including PM₂.₅, NO₂, and O₃, that may be associated with increased AMD risk (16, 17). These exposures may promote inflammation, oxidative stress, and mitochondrial dysfunction, which may damage the RPE and activate pro-angiogenic pathways (16, 17). During periods of severe air pollution, limiting outdoor activity, reducing direct ocular exposure, and using indoor air filtration may be helpful.

## 5 Limitations and strengths

Several limitations should be acknowledged in this review. Only English-language sources were searched, potentially resulting in language bias. Additionally, only published evidence was included, while unpublished or ongoing studies were not considered. The analysis relied exclusively on evidence from prior systematic reviews and meta-analyses; as a result, individual studies excluded from those sources were not considered, potentially limiting the comprehensiveness of the evidence base. In addition, age, which is the most important demographic determinant of AMD, was insufficiently reported in many included meta-analyses. Several reviews did not report a pooled age range, and some primary studies included participants younger than the typical high-risk age group for AMD. Variation in age distribution across studies may have introduced residual confounding and may partly explain the inconsistent findings across risk factors. Although certain limitations exist, this umbrella review offers notable strengths, having integrated evidence from published meta-analyses on AMD risk factors while assessing both the breadth and quality of the available evidence. Independent literature screening, study selection, and data extraction were performed by two reviewers, with methodological quality assessed by AMSTAR and the certainty of evidence evaluated using GRADE.

## 6 Conclusion

This umbrella review demonstrates that AMD risk is associated with lifestyle, systemic diseases, pharmacological exposures, and environmental factors. However, most of the available evidence was classified as low or very low in quality, and only a few associations were supported by moderate-quality evidence. More rigorous studies are needed to clarify these associations.

## Data Availability

The original contributions presented in the study are included in the article/Supplementary material, further inquiries can be directed to the corresponding author.
