# Supplementary material for: Risk factors for age-related macular degeneration: an umbrella analysis of systematic review and meta-analysis
Source: Front Med (Lausanne). 2026 Jun 17;13:1878292. doi: 10.3389/fmed.2026.1878292 (PMC13318758; doi:10.3389/fmed.2026.1878292)
Supplement: Supplementary file 1 [file Supplementary_file_1.docx]

**Supplementary materials**

Table S1. Search strategies used for each database

PubMed (n = 420)

 ((("Macular Degeneration"[Mesh]) OR ((((((((• Age-Related Macular Degeneration) OR (• Age Related Macular Degeneration)) OR (• Age-Related Macular Degenerations)) OR (• Age-Related Maculopathies)) OR (• Age Related Maculopathies)) OR (• Age-Related Maculopathy)) OR (• Age Related Maculopathy)) OR (• AMD))) AND (("Risk Factors"[Mesh]) OR ((((((((((• Risk Factor) OR (• Population at Risk)) OR (• Populations at Risk)) OR (• Risk Scores)) OR (• Risk Score)) OR (• Risk Factor Scores)) OR (• Risk Factor Score)) OR (• Health Correlates)) OR (• Social Risk Factors)) OR (• Social Risk Factor)))) AND (systematic review OR meta-analysis)

Web of Science (n = 363)

(((Macular Degeneration) OR ((((((((• Age-Related Macular Degeneration) OR (• Age Related Macular Degeneration)) OR (• Age-Related Macular Degenerations)) OR (• Age-Related Maculopathies)) OR (• Age Related Maculopathies)) OR (• Age-Related Maculopathy)) OR (• Age Related Maculopathy)) OR (• AMD))) AND ((Risk Factors) OR ((((((((((• Risk Factor) OR (• Population at Risk)) OR (• Populations at Risk)) OR (• Risk Scores)) OR (• Risk Score)) OR (• Risk Factor Scores)) OR (• Risk Factor Score)) OR (• Health Correlates)) OR (• Social Risk Factors)) OR (• Social Risk Factor)))) AND (systematic review OR meta-analysis) (Topic)

Embase (n = 318)


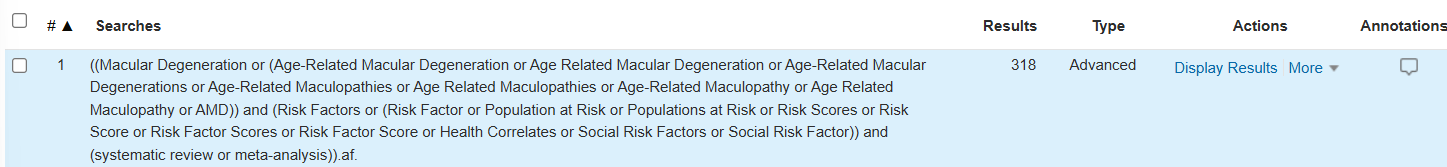


Cochrane (n = 102)


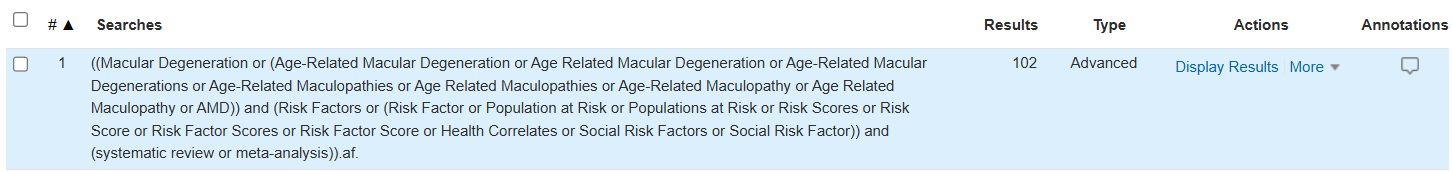


Table S2. Assessments of AMSTAR scores.

| Risk factors | Assessed with | Outcomes | Age range | Included MA | No. of studies T/R/C/P | A priori design provided | Duplicate study selection & data extraction | At least two electronic databases searched | Status of publication used as an inclusion criterion | List of included and excluded studies provided | Characteristics of included studies provided | Scientific quality of included studies assessed | Scientific quality of the included studies used appropriately to form conclusions | Appropriate methods to combine studies | Publication bias assessed | Conflict of interest included | Total AMSTAR Score |
| --- | --- | --- | --- | --- | --- | --- | --- | --- | --- | --- | --- | --- | --- | --- | --- | --- | --- |
| Obstructive Sleep Apnea (OSA) | With vs. without | AMD | NA | Gabriella Bulloch 2024 | 2/0/2/0 | 1 | 1 | 1 | 0 | 1 | 1 | 1 | 0 | 1 | 0 | 1 | 8 |
| C-Reactive Protein Levels | Highest vs. lowest | AMD | NA | Chen Feng 2020 | 49/0/3/46 | 0 | 1 | 1 | 0 | 1 | 1 | 1 | 0 | 1 | 1 | 1 | 8 |
| Dietary omega-3 Polyunsaturated Fatty Acids Intake | Highest vs. lowest | Early AMD | NA | Hong Jiang 2021 | 6/0/3/3 | 1 | 1 | 1 | 0 | 1 | 1 | 1 | 0 | 1 | 1 | 1 | 9 |
| Dietary omega-3 Polyunsaturated Fatty Acids Intake | Highest vs. lowest | Late AMD | NA | Hong Jiang 2021 | 9/1/1/7 | 1 | 1 | 1 | 0 | 1 | 1 | 1 | 0 | 1 | 1 | 1 | 9 |
| Aspirin Usage | With vs. without | AMD | NA | Ruijia Yan 2022 | 16/2/6/8 | 0 | 1 | 1 | 0 | 1 | 1 | 1 | 0 | 1 | 1 | 1 | 8 |
| Cataract Surgery | With vs. without | Late AMD | Study-level only (no overall pooled range) | Lihong Yang 2022 | 5/0/0/5 | 1 | 1 | 1 | 0 | 1 | 1 | 1 | 0 | 1 | 1 | 1 | 9 |
| Cataract Surgery | With vs. without | The progression of AMD | Study-level only (no overall pooled range) | Lihong Yang 2022 | 5/2/0/3 | 1 | 1 | 1 | 0 | 1 | 1 | 1 | 0 | 1 | 1 | 1 | 9 |
| Dementia/AD | With vs. without | AMD | Study-level only (no overall pooled range) | Shi Song Rong 2019 | 3/0/0/3 | 0 | 1 | 1 | 0 | 1 | 1 | 1 | 0 | 1 | 1 | 1 | 8 |
| Mediterranean Diet | With vs. without | AMD | Study-level only (no overall pooled range) | Maria Angelia 2024 | 8/0/5/3 | 0 | 0 | 1 | 0 | 1 | 1 | 1 | 0 | 1 | 0 | 1 | 6 |
| Oral Metformin Use | With vs. without | AMD | Study-level only (no overall pooled range) | Kai-Hsiang Liang 2022 | 9/0/5/4 | 1 | 1 | 1 | 0 | 1 | 1 | 1 | 0 | 1 | 1 | 1 | 9 |
| BMI | Per 1 kg/m^2^ increase in BMI | AMD | NA | Zhang, Q. Y. 2016 | 7/0/7/0 | 0 | 1 | 1 | 0 | 1 | 1 | 1 | 0 | 1 | 1 | 1 | 8 |
| Overweight | Overweight (25–29.9 kg/m^2^) vs. Normal weight (18.5–25 kg/m^2^) | AMD | NA | Zhang, Q. Y. 2016 | 5/0/5/0 | 0 | 1 | 1 | 0 | 1 | 1 | 1 | 0 | 1 | 1 | 1 | 8 |
| Obesity | Obesity (≥30 kg/m^2^) vs. Normal weight (18.5–25 kg/m^2^) | AMD | NA | Zhang, Q. Y. 2016 | 7/0/7/0 | 0 | 1 | 1 | 0 | 1 | 1 | 1 | 0 | 1 | 1 | 1 | 8 |
| Underweight | Underweight (＜18.5 kg/m^2^) vs. Normal weight (18.5–25 kg/m^2^) | AMD | NA | Zhang, Q. Y. 2016 | 4/0/4/0 | 0 | 1 | 1 | 0 | 1 | 1 | 1 | 0 | 1 | 1 | 1 | 8 |
| Hypertension | With vs. without | AMD | Study-level only (no overall pooled range) | Parsa Panahi 2023 | 3/0/0/3 | 0 | 1 | 1 | 0 | 1 | 1 | 1 | 0 | 1 | 1 | 1 | 8 |
| Total Cholesterol (TC) | Per 1 mmol/L increment of TC level | AMD | Study-level only (no overall pooled range) | Wang, Y. 2016 | 18/0/4/14 | 0 | 1 | 1 | 0 | 1 | 1 | 1 | 0 | 1 | 1 | 1 | 8 |
| High-density Lipoprotein Cholesterol (HDL-C) | Per 1 mmol/L increment of HDL-C level | AMD | Study-level only (no overall pooled range) | Wang, Y. 2016 | 15/0/4/11 | 0 | 1 | 1 | 0 | 1 | 1 | 1 | 0 | 1 | 1 | 1 | 8 |
| Low-density Lipoprotein Cholesterol (LDL-C) | Per 1 mmol/L increment of LDL-C level | AMD | Study-level only (no overall pooled range) | Wang, Y. 2016 | 10/0/1/9 | 0 | 1 | 1 | 0 | 1 | 1 | 1 | 0 | 1 | 1 | 1 | 8 |
| Triglycerides (TG) | Per 1 mmol/L increment of TG level | AMD | Study-level only (no overall pooled range) | Wang, Y. 2016 | 9/0/1/8 | 0 | 1 | 1 | 0 | 1 | 1 | 1 | 0 | 1 | 1 | 1 | 8 |
| Thyroid Disease | With vs. without | AMD | NA | Ziming Xu 2021 | 7/0/2/5 | 0 | 1 | 1 | 0 | 1 | 1 | 1 | 0 | 1 | 1 | 1 | 8 |
| Thyroid Medication | With vs. without | AMD | NA | Ziming Xu 2021 | 7/0/3/4 | 0 | 1 | 1 | 0 | 1 | 1 | 1 | 0 | 1 | 1 | 1 | 8 |
| Ambient Air Pollution PM2.5 | With vs. without | AMD | Study-level only (no overall pooled range) | Zhuo Han 2024 | 2/0/0/2 | 0 | 1 | 1 | 0 | 1 | 1 | 1 | 0 | 1 | 1 | 1 | 8 |
| Ambient Nitrogen Dioxide (NO_2_) | With vs. without | AMD | NA | Wu, J. 2024 | 3/0/1/2 | 0 | 1 | 1 | 0 | 1 | 1 | 1 | 0 | 1 | 0 | 1 | 7 |
| Ambient Ozone (O_3_) | With vs. without | AMD | NA | Wu, J. 2024 | 3/0/0/3 | 0 | 1 | 1 | 0 | 1 | 1 | 1 | 0 | 1 | 0 | 1 | 7 |
| Chronic Kidney Disease (CKD) | With vs. without | AMD | Study-level only (no overall pooled range) | Yi-Ju Chen 2018 | 12/0/3/9 | 0 | 1 | 1 | 0 | 1 | 1 | 1 | 0 | 1 | 1 | 1 | 8 |
| Age | Younger vs. older | AMD | Study-level only (no overall pooled range) | Raghad Babaker 2025 | 11/0/5/6 | 1 | 1 | 1 | 0 | 1 | 1 | 1 | 0 | 1 | 1 | 1 | 9 |
| Gender | Male vs. Female | AMD | Study-level only (no overall pooled range) | Raghad Babaker 2025 | 7/0/2/5 | 1 | 1 | 1 | 0 | 1 | 1 | 1 | 0 | 1 | 1 | 1 | 9 |
| Diabetes | With vs. without | AMD | Study-level only (no overall pooled range) | Raghad Babaker 2025 | 6/0/3/3 | 1 | 1 | 1 | 0 | 1 | 1 | 1 | 0 | 1 | 1 | 1 | 9 |
| Cardiovascular Diseases | With vs. without | AMD | Study-level only (no overall pooled range) | Raghad Babaker 2025 | 8/0/4/4 | 1 | 1 | 1 | 0 | 1 | 1 | 1 | 0 | 1 | 1 | 1 | 9 |
| Cerebrovascular Diseases | With vs. without | AMD | Study-level only (no overall pooled range) | Raghad Babaker 2025 | 3/0/1/2 | 1 | 1 | 1 | 0 | 1 | 1 | 1 | 0 | 1 | 1 | 1 | 9 |
| Sunlight Exposure | With vs. without | AMD | NA | Hongjie Zhou 2018 | 14/0/1/13 | 0 | 1 | 1 | 0 | 1 | 1 | 1 | 0 | 1 | 1 | 1 | 8 |
| Vegetables Consumption | Highest vs. lowest | AMD | Study-level only (no overall pooled range) | Monica Dinu 2019 | 4/0/4/0 | 0 | 1 | 1 | 0 | 1 | 1 | 1 | 1 | 0 | 1 | 1 | 8 |
| Fruits Consumption | Highest vs. lowest | AMD | Study-level only (no overall pooled range) | Monica Dinu 2019 | 3/0/3/0 | 0 | 1 | 1 | 0 | 1 | 1 | 1 | 1 | 0 | 1 | 1 | 8 |
| Grain Consumption | Highest vs. lowest | AMD | Study-level only (no overall pooled range) | Monica Dinu 2019 | 2/0/2/0 | 0 | 1 | 1 | 0 | 1 | 1 | 1 | 1 | 0 | 1 | 1 | 8 |
| Nuts Consumption | Highest vs. lowest | AMD | Study-level only (no overall pooled range) | Monica Dinu 2019 | 3/0/3/0 | 0 | 1 | 1 | 0 | 1 | 1 | 1 | 1 | 0 | 1 | 1 | 8 |
| Meat Consumption | Highest vs. lowest | AMD | Study-level only (no overall pooled range) | Monica Dinu 2019 | 6/0/6/0 | 0 | 1 | 1 | 0 | 1 | 1 | 1 | 1 | 0 | 1 | 1 | 8 |
| Dairy Products Consumption | Highest vs. lowest | AMD | Study-level only (no overall pooled range) | Monica Dinu 2019 | 3/0/3/0 | 0 | 1 | 1 | 0 | 1 | 1 | 1 | 1 | 0 | 1 | 1 | 8 |
| Oils Consumption | Highest vs. lowest | AMD | Study-level only (no overall pooled range) | Monica Dinu 2019 | 2/0/2/0 | 0 | 1 | 1 | 0 | 1 | 1 | 1 | 1 | 0 | 1 | 1 | 8 |
| Butter Consumption | Highest vs. lowest | AMD | Study-level only (no overall pooled range) | Monica Dinu 2019 | 2/0/2/0 | 0 | 1 | 1 | 0 | 1 | 1 | 1 | 1 | 0 | 1 | 1 | 8 |
| Margarine Consumption | Highest vs. lowest | AMD | Study-level only (no overall pooled range) | Monica Dinu 2019 | 3/0/3/0 | 0 | 1 | 1 | 0 | 1 | 1 | 1 | 1 | 0 | 1 | 1 | 8 |
| Long Sleep Duration | ＞8h vs. 7-8h | AMD | Study-level only (no overall pooled range) | Miao Zhou 2023 | 2/0/0/2 | 1 | 1 | 1 | 0 | 1 | 1 | 1 | 0 | 1 | 1 | 1 | 9 |
| Short Sleep Duration | ＜7h vs. 7-8h | AMD | Study-level only (no overall pooled range) | Miao Zhou 2023 | 2/0/0/2 | 1 | 1 | 1 | 0 | 1 | 1 | 1 | 0 | 1 | 1 | 1 | 9 |
| Statin Use | With vs. without | AMD | Study-level only (no overall pooled range) | Memarzadeh, E. 2022 | 22/0/11/11 | 0 | 1 | 1 | 0 | 1 | 1 | 1 | 0 | 1 | 1 | 1 | 8 |
| Physical Activity | Sedentary vs. active | Early AMD | Study-level only (no overall pooled range) | McGuinness, M. B.2017 | 8/0/3/5 | 0 | 1 | 1 | 0 | 1 | 1 | 1 | 0 | 1 | 1 | 1 | 8 |
| Physical Activity | Sedentary vs. active | Late AMD | Study-level only (no overall pooled range) | McGuinness, M. B.2017 | 7/0/3/4 | 0 | 1 | 1 | 0 | 1 | 1 | 1 | 0 | 1 | 1 | 1 | 8 |
| Serum Vitamin D levels | Per 10-ng/mL increase in serum vitamin D levels | AMD | Study-level only (no overall pooled range) | Wu, W. 2016 | 8/0/2/6 | 0 | 1 | 1 | 0 | 1 | 1 | 1 | 0 | 1 | 1 | 1 | 8 |
| Smoking | With vs. without | AMD | NA | Asiamah, R. 2025 | 4/0/0/4 | 1 | 1 | 1 | 0 | 1 | 1 | 1 | 0 | 1 | 0 | 1 | 8 |
| Periodontal Disease | With vs. without | AMD | Study-level only (no overall pooled range) | Meng, M. 2024 | 5/0/5/0 | 0 | 1 | 1 | 0 | 1 | 1 | 1 | 0 | 1 | 1 | 1 | 9 |
| Alcohol Consumption | Non/occasional vs. moderate (12–24 g/day) | Early AMD | NA | Jingjing Zhang 2021 | 6/0/5/1 | 0 | 1 | 1 | 0 | 1 | 1 | 1 | 0 | 1 | 0 | 1 | 8 |
| Alcohol Consumption | Non/occasional vs. heavy (≥ 24 g/day) | Early AMD | NA | Jingjing Zhang 2021 | 5/0/5/0 | 0 | 1 | 1 | 0 | 1 | 1 | 1 | 0 | 1 | 0 | 1 | 8 |
| Fish Consumption | With vs. without | AMD | Study-level only (no overall pooled range) | Wei Zhu 2016 | 8/0/8/0 | 0 | 1 | 1 | 0 | 1 | 1 | 1 | 0 | 1 | 1 | 1 | 8 |
| Blood lutein/zeaxanthin Level | Highest vs. lowest | AMD | Study-level only (no overall pooled range) | Jiang, H. 2022 | 9/0/1/8 | 1 | 1 | 1 | 0 | 1 | 1 | 1 | 0 | 1 | 1 | 1 | 9 |
| Blood β-carotene concentrations Level | Highest vs. lowest | AMD | Study-level only (no overall pooled range) | Jiang, H. 2022 | 6/0/1/5 | 1 | 1 | 1 | 0 | 1 | 1 | 1 | 0 | 1 | 1 | 1 | 9 |
| Blood β-cryptoxanthin Level | Highest vs. lowest | AMD | Study-level only (no overall pooled range) | Jiang, H. 2022 | 6/0/1/5 | 1 | 1 | 1 | 0 | 1 | 1 | 1 | 0 | 1 | 1 | 1 | 9 |
| Blood lycopene Level | Highest vs. lowest | AMD | Study-level only (no overall pooled range) | Jiang, H. 2022 | 6/0/1/5 | 1 | 1 | 1 | 0 | 1 | 1 | 1 | 0 | 1 | 1 | 1 | 9 |
| Blood retinol Level | Highest vs. lowest | AMD | Study-level only (no overall pooled range) | Jiang, H. 2022 | 3/0/0/3 | 1 | 1 | 1 | 0 | 1 | 1 | 1 | 0 | 1 | 1 | 1 | 9 |
| Blood α-tocopherol Level | Highest vs. lowest | AMD | Study-level only (no overall pooled range) | Jiang, H. 2022 | 5/0/0/5 | 1 | 1 | 1 | 0 | 1 | 1 | 1 | 0 | 1 | 1 | 1 | 9 |

OSA, Obstructive Sleep Apnea; AD, Alzheimer’s disease; TC, Total Cholesterol; HDL-C, High-density Lipoprotein Cholesterol; LDL-C, Low-density Lipoprotein Cholesterol; TG, Triglycerides; NO2, Ambient Nitrogen Dioxide; O3, Ambient Ozone; CKD, Chronic Kidney Disease; CI, confidence interval; T, total No. of studies; R, randomized controlled trials; C, cohort studies; P, population-based case-control and/or cross-sectional studies; AMSTAR, a measurement tool to assess systematic reviews; GRADE, Grading of Recommendations Assessment, Development, and Evaluation; NA, not available.

Table S3. GRADE classification of quality of evidence.

| Risk factors | Assessed with | Outcomes | Included MA | No. of studies T/R/C/P | Risk of bias | Inconsistency | Indirectness | Imprecision P<0.001 | Publication bias | Plausible confounding | Magnitude of effect | Dose-response gradient | Quality |
| --- | --- | --- | --- | --- | --- | --- | --- | --- | --- | --- | --- | --- | --- |
| Obstructive Sleep Apnea (OSA) | With vs. without | AMD | Gabriella Bulloch 2024 | 2/0/2/0 | Serious risk | Serious inconsistency | No serious indirectness | Serious imprecision | NA | Would not reduce effect | No | No | Very low |
| C-Reactive Protein Levels | Highest vs. lowest | AMD | Chen Feng 2020 | 49/0/3/46 | No serious risk | Serious inconsistency | No serious indirectness | Serious imprecision | Strongly suspected | Would not reduce effect | No | No | Very low |
| Dietary omega-3 Polyunsaturated Fatty Acids Intake | Highest vs. lowest | Early AMD | Hong Jiang 2021 | 6/0/3/3 | No serious risk | No serious inconsistency | No serious indirectness | Serious imprecision | Undetected | Would not reduce effect | No | Yes | Low |
| Dietary omega-3 Polyunsaturated Fatty Acids Intake | Highest vs. lowest | Late AMD | Hong Jiang 2021 | 9/1/1/7 | No serious risk | Serious inconsistency | No serious indirectness | Serious imprecision | Undetected | Would not reduce effect | No | Yes | Very low |
| Aspirin Usage | With vs. without | AMD | Ruijia Yan 2022 | 16/2/6/8 | No serious risk | No serious inconsistency | No serious indirectness | Serious imprecision | Undetected | Would not reduce effect | No | No | Very low |
| Cataract Surgery | With vs. without | Late AMD | Lihong Yang 2022 | 5/0/0/5 | No serious risk | No serious inconsistency | No serious indirectness | Serious imprecision | Undetected | Would not reduce effect | Yes | No | Low |
| Cataract Surgery | With vs. without | The progression of AMD | Lihong Yang 2022 | 5/2/0/3 | No serious risk | No serious inconsistency | No serious indirectness | No serious imprecision | Undetected | Would not reduce effect | No | No | Low |
| Dementia/AD | With vs. without | AMD | Shi Song Rong 2019 | 3/0/0/3 | No serious risk | No serious inconsistency | No serious indirectness | Serious imprecision | Undetected | Would not reduce effect | No | No | Very low |
| Mediterranean Diet | With vs. without | AMD | Maria Angelia 2024 | 8/0/5/3 | No serious risk | Serious inconsistency | No serious indirectness | No serious imprecision | NA | Would not reduce effect | No | No | Low |
| Oral Metformin Use | With vs. without | AMD | Kai-Hsiang Liang 2022 | 9/0/5/4 | No serious risk | Serious inconsistency | No serious indirectness | Serious imprecision | Undetected | Would not reduce effect | No | No | Very low |
| BMI | Per 1 kg/m^2^ increase in BMI | AMD | Zhang, Q. Y. 2016 | 7/0/7/0 | No serious risk | Serious inconsistency | No serious indirectness | No serious imprecision | Undetected | Would not reduce effect | No | Yes | Low |
| Overweight | Overweight (25–29.9 kg/m^2^) vs. Normal weight (18.5–25 kg/m^2^) | AMD | Zhang, Q. Y. 2016 | 5/0/5/0 | No serious risk | No serious inconsistency | No serious indirectness | Serious imprecision | Undetected | Would not reduce effect | No | Yes | Very low |
| Obesity | Obesity (≥30 kg/m^2^) vs. Normal weight (18.5–25 kg/m^2^) | AMD | Zhang, Q. Y. 2016 | 7/0/7/0 | No serious risk | Serious inconsistency | No serious indirectness | Serious imprecision | Undetected | Would not reduce effect | No | Yes | Very low |
| Underweight | Underweight (＜18.5 kg/m^2^) vs. Normal weight (18.5–25 kg/m^2^) | AMD | Zhang, Q. Y. 2016 | 4/0/4/0 | No serious risk | No serious inconsistency | No serious indirectness | Serious imprecision | Undetected | Would not reduce effect | No | Yes | Very low |
| Hypertension | With vs. without | AMD | Parsa Panahi 2023 | 3/0/0/3 | No serious risk | No serious inconsistency | No serious indirectness | Serious imprecision | Undetected | Would not reduce effect | Yes | No | Low |
| Total Cholesterol (TC) | Per 1 mmol/L increment of TC level | AMD | Wang, Y. 2016 | 18/0/4/14 | Serious risk | Serious inconsistency | No serious indirectness | Serious imprecision | Undetected | Would not reduce effect | No | No | Very low |
| High-density Lipoprotein Cholesterol (HDL-C) | Per 1 mmol/L increment of HDL-C level | AMD | Wang, Y. 2016 | 15/0/4/11 | Serious risk | Serious inconsistency | No serious indirectness | Serious imprecision | Undetected | Would not reduce effect | No | No | Very low |
| Low-density Lipoprotein Cholesterol (LDL-C) | Per 1 mmol/L increment of LDL-C level | AMD | Wang, Y. 2016 | 10/0/1/9 | Serious risk | No serious inconsistency | No serious indirectness | Serious imprecision | Undetected | Would not reduce effect | No | No | Very low |
| Triglycerides (TG) | Per 1 mmol/L increment of TG level | AMD | Wang, Y. 2016 | 9/0/1/8 | No serious risk | No serious inconsistency | No serious indirectness | No serious imprecision | Undetected | Would not reduce effect | No | No | Low |
| Thyroid Disease | With vs. without | AMD | Ziming Xu 2021 | 7/0/2/5 | Serious risk | Serious inconsistency | No serious indirectness | Serious imprecision | Undetected | Would not reduce effect | No | No | Very low |
| Thyroid Medication | With vs. without | AMD | Ziming Xu 2021 | 7/0/3/4 | Serious risk | Serious inconsistency | No serious indirectness | Serious imprecision | Undetected | Would not reduce effect | No | No | Very low |
| Ambient Air Pollution PM2.5 | With vs. without | AMD | Zhuo Han 2024 | 2/0/0/2 | No serious risk | No serious inconsistency | No serious indirectness | Serious imprecision | Strongly suspected | Would not reduce effect | Yes | No | Very low |
| Ambient Nitrogen Dioxide (NO_2_) | With vs. without | AMD | Wu, J. 2024 | 3/0/1/2 | No serious risk | Serious inconsistency | No serious indirectness | No serious imprecision | NA | Would not reduce effect | No | No | Very low |
| Ambient Ozone (O_3_) | With vs. without | AMD | Wu, J. 2024 | 3/0/0/3 | No serious risk | Serious inconsistency | No serious indirectness | No serious imprecision | NA | Would not reduce effect | No | No | Very low |
| Chronic Kidney Disease (CKD) | With vs. without | AMD | Yi-Ju Chen 2018 | 12/0/3/9 | No serious risk | Serious inconsistency | No serious indirectness | Serious imprecision | Strongly suspected | Would not reduce effect | No | No | Very low |
| Age | Younger vs. older | AMD | Raghad Babaker 2025 | 11/0/5/6 | No serious risk | Serious inconsistency | No serious indirectness | No serious imprecision | Undetected | Would not reduce effect | No | No | Very low |
| Gender | Male vs. Female | AMD | Raghad Babaker 2025 | 7/0/2/5 | No serious risk | No serious inconsistency | No serious indirectness | Serious imprecision | NA | Would not reduce effect | Yes | No | Low |
| Diabetes | With vs. without | AMD | Raghad Babaker 2025 | 6/0/3/3 | No serious risk | No serious inconsistency | No serious indirectness | No serious imprecision | NA | Would not reduce effect | No | No | Low |
| Cardiovascular Diseases | With vs. without | AMD | Raghad Babaker 2025 | 8/0/4/4 | No serious risk | Serious inconsistency | No serious indirectness | Serious imprecision | NA | Would not reduce effect | No | No | Very low |
| Cerebrovascular Diseases | With vs. without | AMD | Raghad Babaker 2025 | 3/0/1/2 | No serious risk | No serious inconsistency | No serious indirectness | Serious imprecision | NA | Would not reduce effect | No | No | Very low |
| Sunlight Exposure | With vs. without | AMD | Hongjie Zhou 2018 | 14/0/1/13 | No serious risk | No serious inconsistency | No serious indirectness | Serious imprecision | Undetected | Would not reduce effect | No | No | Very low |
| Vegetables Consumption | Highest vs. lowest | AMD | Monica Dinu 2019 | 4/0/4/0 | No serious risk | No serious inconsistency | No serious indirectness | Serious imprecision | NA | Would not reduce effect | No | No | Very low |
| Fruits Consumption | Highest vs. lowest | AMD | Monica Dinu 2019 | 3/0/3/0 | No serious risk | No serious inconsistency | No serious indirectness | Serious imprecision | NA | Would not reduce effect | No | No | Very low |
| Grain Consumption | Highest vs. lowest | AMD | Monica Dinu 2019 | 2/0/2/0 | No serious risk | Serious inconsistency | No serious indirectness | Serious imprecision | NA | Would not reduce effect | No | No | Very low |
| Nuts Consumption | Highest vs. lowest | AMD | Monica Dinu 2019 | 3/0/3/0 | No serious risk | Serious inconsistency | No serious indirectness | Serious imprecision | NA | Would not reduce effect | No | No | Very low |
| Meat Consumption | Highest vs. lowest | AMD | Monica Dinu 2019 | 6/0/6/0 | No serious risk | Serious inconsistency | No serious indirectness | Serious imprecision | NA | Would not reduce effect | No | No | Very low |
| Dairy Products Consumption | Highest vs. lowest | AMD | Monica Dinu 2019 | 3/0/3/0 | No serious risk | Serious inconsistency | No serious indirectness | Serious imprecision | NA | Would not reduce effect | No | No | Very low |
| Oils Consumption | Highest vs. lowest | AMD | Monica Dinu 2019 | 2/0/2/0 | No serious risk | No serious inconsistency | No serious indirectness | Serious imprecision | NA | Would not reduce effect | No | No | Very low |
| Butter Consumption | Highest vs. lowest | AMD | Monica Dinu 2019 | 2/0/2/0 | No serious risk | No serious inconsistency | No serious indirectness | Serious imprecision | NA | Would not reduce effect | No | No | Very low |
| Margarine Consumption | Highest vs. lowest | AMD | Monica Dinu 2019 | 3/0/3/0 | No serious risk | No serious inconsistency | No serious indirectness | Serious imprecision | NA | Would not reduce effect | No | No | Very low |
| Long Sleep Duration | ＞8h vs. 7-8h | AMD | Miao Zhou 2023 | 2/0/0/2 | No serious risk | No serious inconsistency | No serious indirectness | Serious imprecision | Undetected | Would not reduce effect | No | No | Very low |
| Short Sleep Duration | ＜7h vs. 7-8h | AMD | Miao Zhou 2023 | 2/0/0/2 | No serious risk | Serious inconsistency | No serious indirectness | Serious imprecision | Undetected | Would not reduce effect | No | No | Very low |
| Statin Use | With vs. without | AMD | Memarzadeh, E.. 2022 | 22/0/11/11 | Serious risk | Serious inconsistency | No serious indirectness | Serious imprecision | Undetected | Would not reduce effect | No | No | Very low |
| Physical Activity | Sedentary vs. active | Early AMD | McGuinness, M. B.2017 | 8/0/3/5 | No serious risk | No serious inconsistency | No serious indirectness | Serious imprecision | Undetected | Would not reduce effect | No | No | Very low |
| Physical Activity | Sedentary vs. active | Late AMD | McGuinness, M. B.2017 | 7/0/3/4 | No serious risk | No serious inconsistency | No serious indirectness | No serious imprecision | Undetected | Would not reduce effect | Yes | No | Moderate |
| Serum Vitamin D levels | Per 10-ng/mL increase in serum vitamin D levels | AMD | Wu, W. 2016 | 8/0/2/6 | Serious risk | Serious inconsistency | No serious indirectness | Serious imprecision | Undetected | Would not reduce effect | No | No | Very low |
| Smoking | With vs. without | AMD | Asiamah, R. 2025 | 4/0/0/4 | Serious risk | Serious inconsistency | No serious indirectness | No serious imprecision | NA | Would not reduce effect | Yes | No | Very low |
| Periodontal Disease | With vs. without | AMD | Xuewen Lv 2020 | 5/0/1/4 | No serious risk | Serious inconsistency | No serious indirectness | Serious imprecision | Undetected | Would not reduce effect | No | No | Very low |
| Alcohol Consumption | Non/occasional vs. moderate (12–24 g/day) | Early AMD | Jingjing Zhang 2021 | 6/0/5/1 | No serious risk | No serious inconsistency | No serious indirectness | Serious imprecision | Strongly suspected | Would not reduce effect | No | Yes | Very low |
| Alcohol Consumption | Non/occasional vs. heavy (≥ 24 g/day) | Early AMD | Jingjing Zhang 2021 | 5/0/5/0 | No serious risk | No serious inconsistency | No serious indirectness | No serious imprecision | Strongly suspected | Would not reduce effect | No | Yes | Low |
| Fish Consumption | With vs. without | AMD | Wei Zhu 2016 | 8/0/8/0 | No serious risk | No serious inconsistency | No serious indirectness | No serious imprecision | Undetected | Would not reduce effect | No | Yes | Moderate |
| Blood lutein/zeaxanthin Level | Highest vs. lowest | AMD | Jiang, H. 2022 | 9/0/1/8 | No serious risk | No serious inconsistency | No serious indirectness | No serious imprecision | Undetected | Would not reduce effect | Yes | No | Moderate |
| Blood β-carotene concentrations Level | Highest vs. lowest | AMD | Jiang, H. 2022 | 6/0/1/5 | No serious risk | Serious inconsistency | No serious indirectness | Serious imprecision | Undetected | Would not reduce effect | Yes | No | Very low |
| Blood β-cryptoxanthin Level | Highest vs. lowest | AMD | Jiang, H. 2022 | 6/0/1/5 | No serious risk | Serious inconsistency | No serious indirectness | Serious imprecision | Undetected | Would not reduce effect | No | No | Very low |
| Blood lycopene Level | Highest vs. lowest | AMD | Jiang, H. 2022 | 6/0/1/5 | No serious risk | No serious inconsistency | No serious indirectness | Serious imprecision | Undetected | Would not reduce effect | No | No | Very low |
| Blood retinol Level | Highest vs. lowest | AMD | Jiang, H. 2022 | 3/0/0/3 | No serious risk | No serious inconsistency | No serious indirectness | Serious imprecision | Undetected | Would not reduce effect | No | No | Very low |
| Blood α-tocopherol Level | Highest vs. lowest | AMD | Jiang, H. 2022 | 5/0/0/5 | No serious risk | No serious inconsistency | No serious indirectness | Serious imprecision | Undetected | Would not reduce effect | Yes | No | Low |

OSA, Obstructive Sleep Apnea; AD, Alzheimer’s disease; TC, Total Cholesterol; HDL-C, High-density Lipoprotein Cholesterol; LDL-C, Low-density Lipoprotein Cholesterol; TG, Triglycerides; NO2, Ambient Nitrogen Dioxide; O3, Ambient Ozone; CKD, Chronic Kidney Disease; CI, confidence interval; T, total No. of studies; R, randomized controlled trials; C, cohort studies; P, population-based case-control and/or cross-sectional studies; AMSTAR, a measurement tool to assess systematic reviews; GRADE, Grading of Recommendations Assessment, Development, and Evaluation; NA, not available.
